# Plasma levels of OLFM4 in normals and patients with gastrointestinal cancer

**DOI:** 10.1111/jcmm.12679

**Published:** 2015-09-28

**Authors:** Stine N. Clemmensen, Anders J. Glenthøj, Sara Heebøll, Hans Jørgen Nielsen, Claus Koch, Niels Borregaard

**Affiliations:** ^1^ The Granulocyte Research LaboratoryDepartment of HematologyNational University HospitalCopenhagenDenmark; ^2^ Department of PathologyNational University HospitalCopenhagenDenmark; ^3^ Department of Hepatology and GastroenterologyAarhus University HospitalAarhusDenmark; ^4^ Department of Surgical GastroenterologyHvidovre HospitalHvidovreDenmark; ^5^ Department of BiomedicineUniversity of Southern DenmarkOdenseDenmark

**Keywords:** olfactomedin 4, colorectal cancer, intestinal stem cells, neutrophil subsets, monoclonal antibodies

## Abstract

Olfactomedin 4 (OLFM4) is a secreted glycoprotein predominantly expressed in bone marrow and gastrointestinal tissues. Aberrant expression of OLFM4 has been shown in several cancers. However, the clinical significance hereof is currently controversial. OLFM4 has been proposed as a candidate biomarker of gastrointestinal cancers. To address this, we developed monoclonal antibodies against synthetic peptides representing various segments of OLFM4. We examined expression of OLFM4 in epithelial cells by immunohistochemistry and found that OLFM4 is highly expressed in proliferating benign epithelial cells and in some carcinoma cells. We developed an Enzyme Linked Immunosorbent Assay for OLFM4 and investigated whether plasma levels of OLFM4 reflect colorectal malignancies, but were unable to see any such association. Instead, we observed two populations of individuals with respect to OLFM4 levels in plasma, the majority with OLFM4 in plasma between 0 and 0.1 μg/ml, mean 0.028 μg/ml while 10% of both normals and patients with cancers had OLFM4 between 4 and 60 μg/ml, mean 15 μg/ml. The levels were constant over time. The background for this high plasma level is not known, but must be taken into account if OLFM4 is used as biomarker for GI cancers.

## Introduction

Olfactomedin 4 (OLFM4) is a highly glycosylated protein. The OLFM4 gene was initially cloned from human hematopoietic stem cells induced to differentiate *in vitro* with G‐CSF, hence the name hGC‐1 (Human Granulocyte Colony Stimulating Factor Stimulated Clone‐1) 1. The gene had previously been identified as GW112, a gene highly expressed in colon cancers (Unigene ID#Hs.273321) and the protein was identified as pDP4 (PU.1 difference product 4), a glycoprotein secreted by mature murine granulocytes and induced at a late stage of maturation by the myeloid specific transcription factor PU.1 2.

We recently identified OLFM4 as a protein localized to specific granules of human neutrophils, but present only in a subset of these, ranging between 5% and 40% of neutrophils between individuals, but constant in each individual over time 3; a finding that was recently confirmed by others 4. The function of OLFM4 in myeloid cells is unknown. It has been suggested to exert anti‐apoptotic activities 5 and to be localized to mitochondria 6. Olfactomedin 4 has been shown to inhibit cathepsin C (also known as DPPI) 7, 8, a cysteine protease localized to azurophil granules of human neutrophils 9 and essential for activation of serine proteases 10. These observations on localization and function of OLFM4 are difficult to reconcile with localization of OLFM4 in specific granules 3.

Olfactomedin 4 is also produced by epithelial cells. It is a marker of epithelial stem cells in the large and small intestines in man 11, but restricted to Lgr5 positive stem cells of small intestines in mice 12. It is up‐regulated in inflammatory bowel disorders 13, which is consistent with the known induction by NF‐κB 14, 15. It is expressed in proliferating human endometrium in response to both 17β‐estradiol and epidermal growth factor 16. As mentioned above, OLFM4 was identified as a gene highly up‐regulated in cancers of the gastrointestinal tract. However, the association is not straight forward. While OLFM4 overexpression has been observed in some cancers, not only of the intestines, but also in lung cancer, gastric cancer and breast cancer 17, others find OLFM4 expressed in differentiated, but not undifferentiated gastric cancer cells 18. In addition, OLFM4 has been reported inhibitory to the growth and metastasis of prostate cancer 19 and malignant melanoma 20, suggesting that the behaviour of OLFM4 is tissue specific or cancer type specific.

Cancers of colon and rectum are common in the industrialized world. Identification of biomarkers that may be used as screening for early stage colorectal cancers is therefore highly desirable. As a secreted glycoprotein, OLFM4 is an obvious candidate biomarker. Mass spectrometry used to analyse cellular proteomes including secretomes, identified OLFM4 as a candidate biomarker in colorectal cancer 21, 22. In accordance, a Japanese study found significantly higher plasma levels of OLFM4 in 123 gastric cancer patients compared to healthy controls 23. However, the level of OLFM4 in plasma has not yet been tested as biomarker for patients diagnosed with primary colorectal cancer.

We decided to generate monoclonal antibodies against selected synthetic peptides, representing unique parts of the OLFM4 protein, to verify their ability to detect OLFM4 both in neutrophils and in epithelial cells, and to develop an ELISA for OLFM4 that would allow quantification of OLFM4 in subcellular fractions of neutrophils and in plasma, and to test, whether levels in plasma reflect malignancies of the gastrointestinal tract.

## Materials and methods

### Generation of monoclonal antibodies

An N‐terminal 21‐aa synthetic peptide (DLGDVGGIPSPGFSSFPGVDSC), a C‐terminal 30‐aa synthetic peptide, CNYNPFDQKLYUVYNDGYLLNYDLSVLQKPQ and a combined synthetic peptide (AWGRDYSPQHPNKGLYKDQNTPVVHPPPTPGSSRSGSSSSRSLGSGGS KDQNTPVVHPPPTPGS) composed of aa sequences (aa #273‐288, aa #50‐65, aa #230‐245) were used for immunizations. A cysteine was added to the C‐terminal of the N‐terminal sequence and to the N‐terminal of the C‐terminal sequence, and both were coupled onto diphtheria toxoid as carrier protein using EMCS as coupling reagent. The sequences in the combined peptide were selected using an algorithm based on surface exposure, the presence of β‐turns and the likelihood of sequences that might be exposed as linear epitopes. The sequences were combined to minimize the presence of terminal sequences. The carrier coupled peptide and the combined peptide (obtained commercially from Schaeffer) were adsorbed onto Al(OH)_3_ and finally mixed in a 1:1 ratio with incomplete Freund's adjuvant and used for generation of monoclonal antibodies. Female NMRI‐mice, 8–10 weeks old, were immunized three times with 25 μg of the individual peptides and sera were tested in an ELISA‐assay against the immunizing peptide coupled onto ovalbumin. Three days prior to fusion, the mice received an intravenous injection in the tail vein with 25 μg of antigen together with adrenalin. The fusion and selection were done essentially as described by Kohler and Milstein 24. The SP2/0‐AG14 myeloma cell line was used as fusion partner. Positive clones were selected by screening against the peptides coupled to ovalbumin. Cloning was performed by limiting dilution. Individual clones were grown in culture flasks in RPMI (Roswell Park Memorial Institute) + 10% FCS and monoclonal antibodies were purified from culture supernatants by Protein A affinity chromatography. Hybridoma supernatants were screened by their ability to react with the antigens used for immunization. Positive clones were then screened based on their ability to react with the content of isolated specific granules of human neutrophils, previously shown to contain OLFM4 3. Two clones were selected based on the ability to give a positive read out during screening and by immunohistochemistry in cytospins of purified human neutrophils. Clone #40 recognized the peptide AWGRDYSPQHPNKGLY (aa #273‐288) and clone #49 recognized the N‐terminal peptide. Cytospins were generated, fixed and stained as described 3.

### Subcellular fractionation of neutrophils

Neutrophils were isolated either from buffy coats supplied by the blood bank and transfusion service at Rigshospitalet or from healthy volunteers giving written informed consent. Blood or buffy coats were mixed with 2% w/v Dextran T‐500 (Sigma‐Aldrich, St. Louis, MO, USA) in saline for sedimentation of erythrocytes at room temperature. Mononuclear cells (MNCs) were separated from neutrophils by density centrifugation on Lymphoprep (Axis‐shield, Oslo, Norway) and residual erythrocytes removed by hypotonic lysis as described 25.

Isolated neutrophils were treated with 5 μM di‐isopropyl fluorophosphates (Sigma‐Aldrich) for inactivation of serine proteases and cavitated as described 25. Nuclei and un‐broken cells were sedimented by centrifugation and the supernatant was layered atop of a 3‐layer Percoll density gradient and subjected to high‐speed centrifugation as described 25, 26. Fractions of 1 ml each were collected and analysed for markers of organelles: Myeloperoxidase (azurophil granules), Neutrophil Gelatinase Associated Lipocalin (NGAL) (specific granules), Gelatinase (gelatinase granules), Albumin (secretory vesicles) and human leucocyte antigen (plasma membranes) as described in 27. Percoll was removed from fractions by ultracentrifugation as described 25. A 4‐layer Percoll density gradient was used for demonstrating the subcellular localization of OLFM4 in neutrophils 28.

### Purification of OLFM4

Specific granules isolated by subcellular fractionation of buffy coat neutrophils were retrieved from Percoll by ultracentrifugation 25 and lysed in PBS, 0.2% Triton X‐100. Undissolved material was removed by high‐speed centrifugation. The supernatant was collected and high molecular weight proteins were precipitated by 15% ammonium sulphate. The pellet was resuspended in PBS and applied to a CNBr‐activated Sepharose column (Pharmacia, Uppsala, Sweden) to which 5 mg monoclonal antibody clone #49 had been coupled according to instructions provided by the manufacturer. The column was washed with PBS followed by 200 mM glycine, pH 2.5 and then eluted with 50 mM tri‐ethanolamine containing 150 mM NaCl, pH 11.5. The eluates were neutralized by HCl and pooled. High molecular weight proteins were again precipitated by 15% ammonium sulphate. A sample of the precipitate was sent to Alphalyse for identification by mass spectrometry.

### Mass Spectrometry

The sample was reduced and alkylated with iodoacetamide and digested with trypsin. The resulting peptides were concentrated in a ZipTip micropurification column and eluted onto an AnchorChip target for analysis on a Bruker Autoflex Speed MALDI TOF/TOF instrument. The peptide mixture was analysed in a positive reflector mode for accurate peptide mass determination. Maldi MS/MS was performed on 15 peptides for peptide fragmentation analysis. The MS and MS/MS spectra were combined and used for database searching using the Mascot software.

### Size exclusion chromatography

Specific granules were isolated as described above 25. Percoll was removed by ultracentrifugation and the specific granules resuspended in 5 ml ice‐cold 100 mM saline, 10 mM Tris, pH 7.6 to which Triton X‐114 was added to a final concentration of 0.7% vol./vol. Phase separation between hydrophobic and hydrophilic phases was then induced 29. The Triton‐poor supernatant containing OLFM4 and other hydrophilic granule proteins was concentrated by filtration (Amicon, Merck Millipore, Darmstadt, Germany) and 200 μl was applied on a Superose 12 column using the FPLC system (Pharmacia). Fractions of 1 ml were sampled. Blue dextran (MW2000 kD) in 200 μl and 200 μl plasma were applied in separate runs.

### Immunohistochemistry

Immunohistochemistry was performed on archival paraffin embedded human tissue obtained from normal colon, duodenum, lung, thymus, liver, spleen. Six needle biopsies from the prostate gland were included. Two needle biopsies contained only malignant tissue, while the remaining four contained both benign tissue and adenocarcinoma. Gleason score was between 6 and 9. Furthermore, different subtypes of colonic adenocarcinomas and colonic adenomas/precursor lesions were included. Tissue material had been fixed in buffered formalin and embedded in paraffin. Three micron sections were made. Immunochemical staining was done using the following murine antibodies as primary antibody OLFM4 #40 (0.48 μg/ml), OLFM4 #49 (0.70 μg/ml). The sections were pre‐treated in PT Link (Dako, Glostrup, Denmark) using high pH target retrieval solution (DM828; Dako) and stained in a DakoLink 48 (Dako) utilizing the Envision Flex+ detection kit (K8002; Dako). Primary antibodies were diluted in Antibody Diluent (DM830; Dako) and incubated for 20 min. The sections were counterstained with haematoxylin (Dako) and analysed using an Olympus BX51 microscope (PlanApo 20x/0.70 objective) with an Olympus UC30 camera and the Olympus cellSens software package (Olympus, Ballerup, Denmark).

### ELISA

A sandwich ELISA was established using the anti‐OLFM4 monoclonal antibodies selected. 96‐well flat‐bottom immunoplates (Nunc, Roskilde, Denmark) were coated overnight with anti‐OLFM4 #49 (0.70 μg/ml) diluted 1:100 in carbonate buffer (50 mM Na_2_CO_3_/NaHCO_3_, pH 9.6). The plates were washed three times in washing buffer (0.5 M NaCl, 3 mM KCl, 8 mM Na_2_HPO_4_/KH_2_PO_4_, 1% Triton X‐100, pH 7.2) and blocked by incubation 1 hr in dilution buffer (0.5 M NaCl, 3 mM KCl, 8 mM Na_2_HPO_4_/KH_2_PO_4_, 1% BSA, 1% Triton X‐100, pH 7.2) 200 μl/well. All subsequent additions were made in 100 μl/well, and incubations were for 1 hr followed by washing three times. Samples and standards (purified neutrophil OLFM4 ranging from 0.022 to 1.4 μg/ml) were applied, followed by incubation with biotinylated anti‐OLFM4 #40 (0.48 μg/ml) antibodies (1:200). Avidin/HRP (Dako) was finally added at a 1:3000 dilution in dilution buffer. Colour was developed by a 30 min. incubation in substrate buffer (0.1 M sodium phosphate, 0.1 M citric acid, pH 5.0) containing 0.04% OPD (Ortho‐Phenylenediamine dihydrochloride) (Dako) and 0.006% H_2_O_2_. The reaction was stopped by adding 100 μl/well 1 M H_2_SO_4_. Absorbance was read at 492 nm in a Synergy 2 multi‐mode reader (BioTek, United States).

Ethylenediaminetetraacetic acid plasma samples were taken from consenting healthy volunteers associated with the institution or selected from the Endoscopy II study, which included 4698 subjects offered first time ever complete colonoscopy because of the symptoms of colo‐rectal cancer (CRC). The samples were collected according to a validated standard operating procedure (SOP) and stored at −80°C under 24/7 electronic surveillance. The plasma included samples from age and gender matched patients with CRC, adenoma and subjects without any large bowel pathology (no neoplastic findings, no diverticulosis and no signs of inflammatory bowel disease). The study has been approved by the Ethics Committee of the Capital Region of Denmark, permissions no. H‐3‐2009‐110 and H‐1‐2011‐165.

### Western blotting

Gel electrophoresis and western blotting were performed by standard procedures on 1.5 mm thick, 12% polyacrylamide gels and blotting to nitrocellulose membranes.

### Biosynthesis

Bone marrows were aspirated from healthy volunteers. Fifteen ml marrow was withdrawn into a 20 ml syringe containing 5 ml ACD and mixed with equal volume of 2% Dextran T‐500 in saline to sediment erythrocytes. The cell rich supernatant was underlayered with Lymphoprep and centrifuged to separate immature myeloid cells (MNCs) and mature myeloid cells (PMNs). Each population was suspended at 2.0 × 10^7^ cells/ml in methionine/cysteine free medium PMI 1640 (Gibco Life Technologies) and incubated for 30 min. at 37°C. The cells were pelleted and resuspended at 3.0 × 10^7^ cells/ml in identical medium to which ^35^S methionine/cysteine (EasyTag; PerkinElmer) was added to a final concentration of 200 μCi/ml. The cells were incubated for 60 min. at 37°C, washed twice in RPMI and resuspended at 2 × 10^7^ cells/ml in RPMI, containing 10% dialysed foetal calf serum, penicillin and streptomycin and divided into 2 equal aliquots that were incubated for 4 and 18 hrs at 37°C, respectively. After centrifugation, the cells were lysed at 10^7^ cells/ml in RIPA buffer (150 mM NaCl, 1% [v/v] Triton X‐100, 0.1% [w/v] SDS, 1% [w/v] sodium deoxycholate, 30 mM HEPES (4‐(2‐hydroxyethyl)‐1‐piperazineethanesulfonic acid), pH 7.3) containing protease inhibitors (complete mini; Roche), 1 tablet/10 ml RIPA buffer, 1 mM phenylmethylsulphonyl fluoride (Sigma‐Aldrich), 200 KIE/ml aprotinin (Trasylol; Bayer, Germany), and 100 mg/ml leupeptin (Sigma‐Aldrich). The samples were incubated on ice for 2 hrs and cleared by centrifugation at 10,000 × g for 30 min. The supernatant was aspirated and subjected to immunoprecipitation. Likewise, the medium from the chase was subjected to immune precipitation. SDS‐sample buffer was added and the samples run on 12% polyacrylamide gels. The gels were stained with Coomassie, destained, incubated with Amplifier (GE Healthcare, United States), dried, and developed on Fuji BAS2500 PhosphoImager.

### Hepatic OLFM4 mRNA expression

Liver biopsies were obtained from adult patients suspected of non‐alcoholic fatty liver disease at Aarhus University Hospital, Aarhus, Denmark with approval from the Danish National Committee on Health Research Ethics (# 20110132). In short, a percutaneous liver biopsy was obtained with a Menghini biopsy needle inserted in intercostal space 8 or 9 in the mid‐axillary line. RNA was extracted from liver biopsies using TRIzol Reagent (Life Technologies Inc., Carlsbad, CA, USA). RNA was quantified by measuring absorbance at 260 and 280 nm using a NanoDrop 8000 (NanoDrop Products, Wilmington, DE, USA). The integrity of the RNA was checked using Bioanalyzer 2100 (Agilent, CA, USA). Total RNA (100 ng) was labelled with the Ambion WT Expression Kit (Ambion, CA, USA), according to the manufacturer's instructions. Samples were hybridized overnight to the GeneChip^®^ Human Gene 2.0 ST Array (Affymetrix Inc., CA, USA), containing 48,000 probes and scanned using an Affymetrix GCS 3000 7G scanner.

## Results

**Figure 1:**
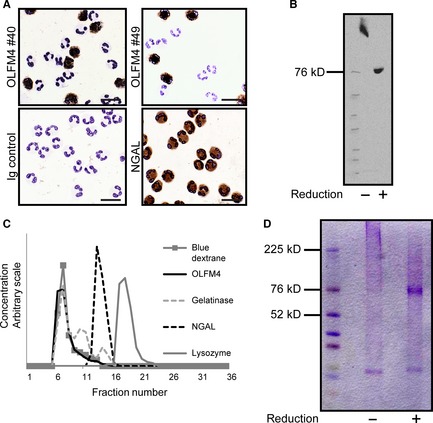
Identification of OLFM4 in Neutrophils. (**A**) Cytospins of neutrophils isolated from peripheral blood were immune‐stained using OLFM4 antibodies from clone #40 and #49. Clearly only a fraction of neutrophils stain positive for OLFM4 whereas all neutrophils stain positive for NGAL. (**B**) Western blotting of isolated specific granules using anti‐OLFM4 antibody #49 under reducing and non‐reducing conditions. (**C**) Size exclusion chromatography of OLFM4 from specific granules of human neutrophils. Distribution of markers was determined by ELISA or by absorption of light (Blue Dextran). (**D**) Coomassie blue stained gels of affinity purified OLFM4 under reducing and non‐reducing conditions.

We first tested whether the monoclonal antibodies were able to react with human neutrophils in immunohistochemistry as demonstrated in Figure 1A. Notably, both monoclonal antibodies stain only a subset of neutrophils as observed previously using both commercial monoclonal antibodies and an in‐house rabbit antibody, generated against another synthetic peptide 3.

Western blotting of isolated specific granules from human neutrophils demonstrated immunoreactivity against an antigen of 72 kD under reducing conditions and above 200 kD in unreduced samples (Fig. 1B), indicating that the antigen is a multimer because of intermolecular disulphide bonding as described previously for recombinant OLFM4 30. The high molecular weight form of native OLFM4 was confirmed by size exclusion chromatography on Superose 12, where OLFM4 was detected using the ELISA described further below (Fig. 1C).

To make a definitive identification of the antigen as OLFM4, the antigen was isolated by affinity chromatography on a CNBr‐activated Sepharose column to which antibody from clone #49 had been coupled. The eluted material was run on SDS‐PAGE under reducing conditions and the part of the gel, corresponding to localization of OLFM4 as determined by western blotting on a gel run in parallel, was cut and subjected to identification by mass spectrometry. The sequence: GFSYLY GAWGRDYSPQ HPNKGLYWVA PLNTDGRLLE YYRLYNTLDD LLLYINAR was obtained. This definitively identifies the antigen as OLFM4.

Affinity purified OLFM4 from specific granules was subjected to SDS‐PAGE under reducing and non‐reducing conditions. Protein staining by Coomassie brilliant blue is given in Figure 1D. The diffuse high MW band seen under non‐reducing conditions is converted to a narrow band at 72 kD upon reduction. As no additional bands appear upon reduction, we conclude that the high molecular weight form of OLFM4 is due to homotypic multimerization by disulphide bonding. Indeed, there are unpaired cysteines in the OLFM4 structure that would allow for such 31.

To ascertain whether the monoclonal antibodies recognize OLFM4 expressed in epithelial cells, the two monoclonal antibodies, clone #40 and clone #49, respectively, were applied to archival formalin fixed tissue. They gave essentially the same staining, but clone #49 could be used in higher dilution (1:1000), and results with this are presented.

**Figure 2:**
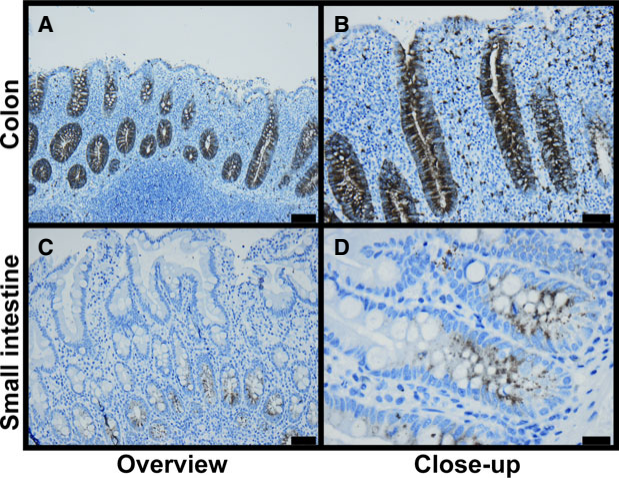
Immunohistochemistry of Human Gastrointestinal Epithelial Cells. Anti‐OLFM4 clone #49 was used as primary antibody. The sections were counterstained with haematoxylin. (**A** and **B**) Colon. (**C** and **D**) Duodenum. Bars represent 50 μm (**A**,** C**) or 20 μm (**B**,** D**).

A distinct staining was observed in crypt cells of the intestines corresponding to the previous reported localization in epithelial stem cells (Fig. 2A). Of note, the staining in crypt cells was uniform in contrast to the staining of peripheral blood neutrophils where only a subset of neutrophils stain positive. The staining was strongest in the proliferating cells at the bottom of the crypt and absent at the surface. Pneumocytes were faintly positive (Fig. S1A and B). Thymus was negative except for a distinct staining of the corpuscles of Hassall (Fig. S1C and D). A very weak punctate staining was observed in hepatocytes. This was considered an unspecific reaction (Fig. S1E and F). The red pulp of the spleen contained OLFM4 positive as well as OLFM4 negative granulocytes in a ratio corresponding to the one observed in peripheral blood polymorphnuclear cells (PMN), the splenocytes themselves did not stain (Fig. S1G and H). No staining was observed in six prostatic adenocarcinomas, whereas staining was observed in benign prostatic epithelium (Fig. S2).

We next examined staining of colon carcinoma cells in different histopathological subtypes of large bowel malignancies, all shown in Figure S3. The staining is highly variable, ranging from negative to uniform brisk positive staining. Some samples show intratumoral heterogeneity. We hypothesized that the areas with vigorous OLFM4 staining could be areas associated with high proliferative potential and therefore stained for ki67. This, however, revealed a homogenous expression of ki67 (Fig. S3P).

**Figure 3:**
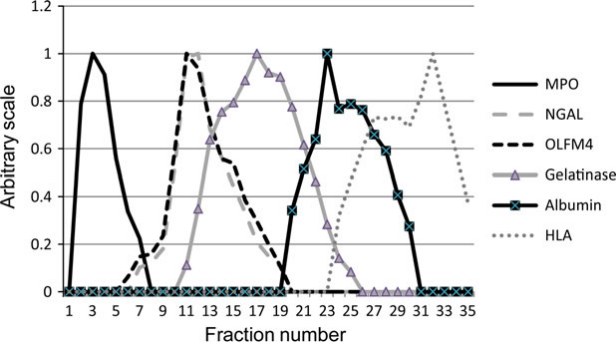
Subcellular Distribution of OLFM4 in Neutrophils. Neutrophils (4.8 × 108) isolated from peripheral blood were cavitated in 12 ml disruption buffer and 5 ml of the post‐nuclear supernatant applied to each of two 4‐layer Percoll density gradients. Separation of organelles was obtained by high‐speed centrifugation and 1 ml fractions were collected from the bottom of the centrifuge tube, pooled from the two identical gradients and assayed for myeloperoxidase (MPO; azurophil granules), NGAL (specific granules), Gelatinase (gelatinase granules), Albumin (secretory vesicles) and human leucocyte antigen (HLA; plasma membranes) and OLFM4. The distribution profile of OLFM4 is identical to the profile of the specific granule protein NGAL.

**Figure 4:**
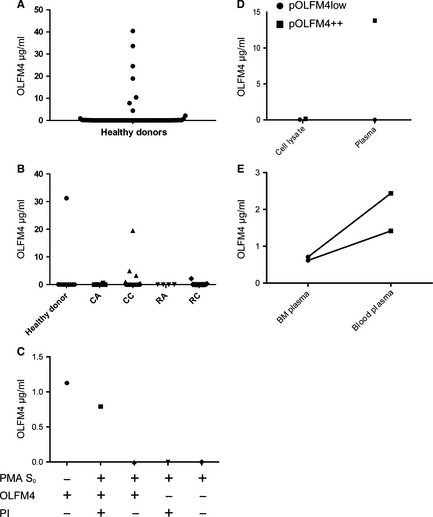
OLFM4 in plasma. (**A**) Plasma samples were obtained from healthy donors (*N* = 65) OLFM4 plasma concentration was measured by ELISA. (**B**) Plasma samples from patients offered first time ever colonoscopy. Depending on the endoscopy finding patients were divided into five groups: No neoplastic finding (*N* = 10), colon adenoma (CA) (*N* = 6), colon carcinoma (CC) (*N* = 23), rectal adenoma (RA) (*N* = 4) and rectal carcinoma (RC) (*N* = 16). OLFM4 plasma concentration was measured by ELISA. Baseline characteristics of patients are presented in Table S1. (**C**) Purified OLFM4 was added to either buffer or medium from neutrophils (10^8^ cells/ml) stimulated to degranulate by stimulation with PMA 5 μg/ml (PMA S_0_) in the presence or absence of protease inhibitors (PI). OLFM4 concentration was measured by ELISA. (**D**) OLFM4 content in cell lysates (1 × 10^7^ cells/ml) and plasma was measured by ELISA in an individual with low plasma OLFM4 (pOLFM4low) and in an individual with high plasma OLFM4 (pOLFM4++). (**E**) ELISA measurements of OLFM4 content in bone marrow (BM) plasma and blood plasma.

Having demonstrated that the monoclonal antibody is specific for OLFM4 and recognizes OLFM4 both in normal epithelial crypt cells and in colon cancer cells, we made a sandwich ELISA for OLFM4 based on the monoclonal antibody from clone #49 as catching antibody and the monoclonal antibody from clone #40 as detecting antibody. The ELISA detected OLFM4 in specific granules of human neutrophils, showing a perfect co‐localization with another recognized marker of specific granules, NGAL 32 (Fig. 3). The ELISA was robust giving an inter assay variation coefficient (S.D./mean ratio) of 5.9% for plasma OLFM4 (*N* = 11) and 4.5% for OLFM4 isolated from β‐band (*N* = 11) and an intra assay variation of 4.6% (*N* = 5). The assay was applied to human plasma and demonstrated an excellent recovery of 97.5% of OLFM4 added to plasma, indicating that OLFM4 in plasma is available for detection by the ELISA. We first tested 65 healthy volunteers associated with the Institution. While most individuals had plasma OLFM4 levels below 100 ng/ml, approximately 10% had levels that were 2–4 orders of magnitude higher (Fig. 4A). We subsequently tested plasma samples selected from the Endoscopy II study on participants undergoing first time ever colonoscopy because of symptoms of CRC 33. The selection included age and gender matched patients with CRC, adenoma and participants without any bowel lesions. Again we identified 10% of patients to have an extremely high level of plasma OLFM4 with no correlation with malignancy (Fig. 4B). We thus conclude that OLFM4 levels in plasma do not reflect OLFM4 expression by colorectal cancers and that OLFM4 identifies a subset of normal individuals with an extremely high level of plasma OLFM4.

One such was identified among healthy volunteer blood and bone marrow donors associated with the Granulocyte Research Laboratory. Repeated testing through 1 year demonstrated that the high OLFM4 plasma level was maintained and did not relate to the size of the OLFM4 positive neutrophil subset detected in peripheral blood from the same person, which was only in the range of 3–5% OLFM4 positive neutrophils.

We have shown previously that OLFM4 is secreted from PMA stimulated neutrophils in parallel with other specific granule proteins 3. When purified OLFM4 was added to medium from neutrophils induced to degranulate with PMA, the ability to detect OLFM4 was rapidly lost, indicating that OLFM4 is highly sensitive to proteolysis. Adding a cocktail of protease inhibitors to the material secreted from PMA activated neutrophils preserved OLFM4 (Fig. 4C).

It is not likely that OLFM4 present in plasma is derived from neutrophil degranulation in blood, as the amounts of OLFM4 in neutrophils do not correlate with the levels in plasma (Fig. 4D). We next determined whether OLFM4 might be produced by bone marrow cells and released into bone marrow plasma. Corresponding levels of OLFM4 determined in bone marrow plasma and blood plasma from two persons with high levels of OLFM4 showed lower levels in bone marrow plasma than in blood plasma, arguing against bone marrow as the direct source of OLFM4 in plasma (Fig. 4E).

**Figure 5:**
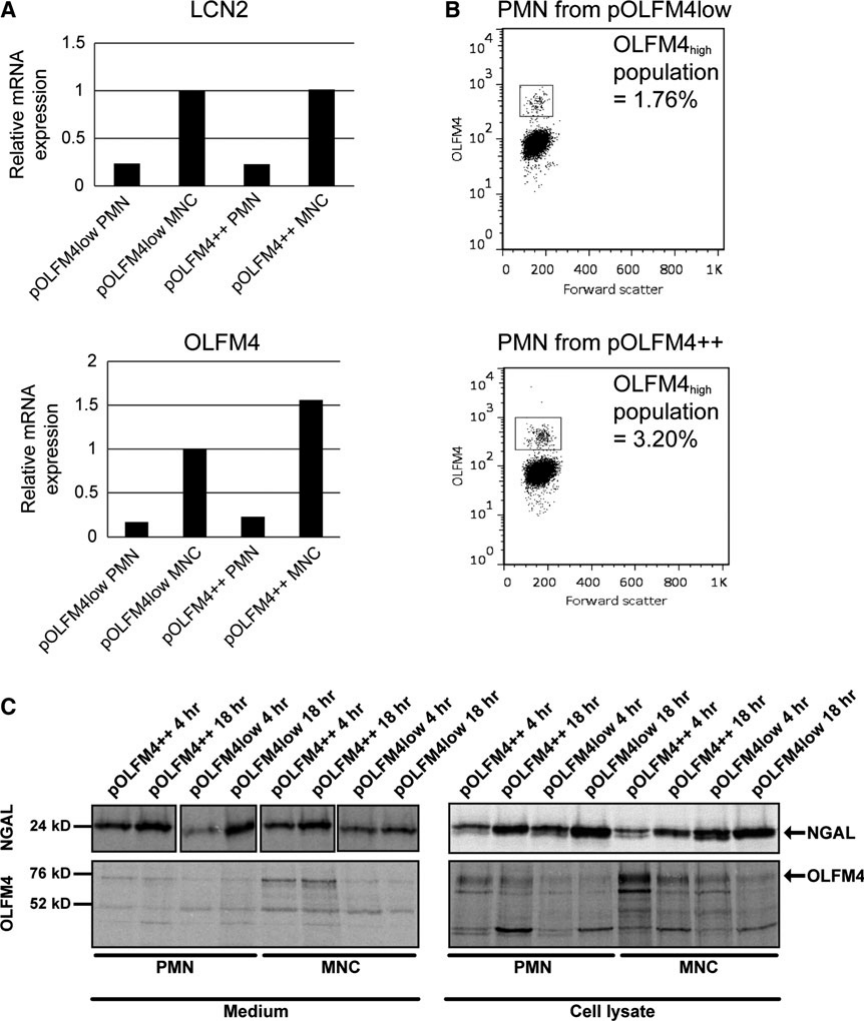
OLFM4 production in an individual with high OLFM4 plasma level (pOLFM4++) and an individual with OLFM4 plasma level below detection limit (pOLFM4low). (**A**) mRNA expression of *OLFM4* and *LCN2* in MNC fraction of bone marrow (containing promyelocytes, myelocytes and early metamyelocytes) and PMN fraction (containing late metamyelocytes, band cells and segmented cells). (**B**) Flowcytometry showing the percentage of OLFM4 positive cells in peripheral blood PMNs from pOLFM4++ person and pOLFM4low person respectively. (**C**) Immunoprecipitated radiolabelled OLFM4 (precipitated using a combination of mouse anti‐human OLFM4 clone #40, clone#49 and rabbit antihuman OLFM4 clone #3569) and NGAL from bone marrow MNC (2 × 10^7^ cells from each donor) and PMN (7.2 × 10^7^ cells from each donor) fractions. The production of OLFM4 in cells from the pOLFM4++ person is comparable to the production in the pOLFM4low individual, the small difference is due to the lower proportion of OLFM4 positive cells in the plasma OLFM4 low person as shown in (**B**).

We next performed studies on the biosynthesis of OLFM4 in isolated bone marrow cells from an individual with high plasma OLFM4 and an individual with low plasma OLFM4. The levels of OLFM4 mRNA were of comparable size (Fig. 5A). The small difference is due to the lower proportion of OLFM4 positive cells in the plasma OLFM4 low person (Fig. 5B). The amounts of OLFM4 synthesized and retained in cells during a 4 hr and an 18 hr chase were quite similar between the two individuals. Only little OLFM4 was detected in medium (Fig. 5C). Thus, these results did not reveal any difference in the amounts of OLFM4 synthesized and retained in neutrophils of a person with high plasma OLFM4 compared to a person with low plasma OLFM4.

To estimate the amount of OLFM4 generated daily during myelopoiesis, we quantified the amount of OLFM4 in neutrophils from 3 sets of buffy coat neutrophils, each pooled from 4 healthy donors. The amount of OLFM4 was 1.2 μg/10^7^ neutrophils. As the production of neutrophils is about 1 × 10^9^ cells/kg bodyweight/day 34, this would indicate production of 10 mg OLFM4/day in an adult.

To rule out the liver as a production site, mRNA was determined by Affymetrix gene array in liver biopsies from 42 patients evaluated for liver steatosis. OLFM4 mRNA levels were uniformly at the border of detection in all (data not shown). Consistent with this, there was no immunohistochemical staining of OLFM4 in normal liver tissue in 16 biopsies, altogether arguing against the liver as producer of OLFM4 (data not shown).

## Discussion

Olfactomedin 4 is an enigmatic protein. While uniformly expressed in epithelial gastrointestinal stem cells, it is expressed in neutrophils at the myelocyte/metamyelocyte stage, *i.e*. in cells that are no longer dividing. Furthermore, OLFM4 is expressed in only a subset of these 3. Clearly, the regulation of expression in epithelial cells and in mesenchymal cells is very different. While the expression in murine neutrophils is induced by PU.1, this transcription factor is myeloid specific 35 and cannot account for the expression in epithelial crypt cells.

Olfactomedin 4 is associated with some cancers, but we did not find a uniform expression that in any way may serve as a marker of malignancy. Much to our surprise, we found plasma levels of OLFM4 covering a wide range, but largely segregating both normals and cancer patients into two groups, one with OLFM4 levels that are barely detectable and one with levels that are 2–4 orders of magnitude higher. We have no evidence that the low levels are caused by masking the presence of OLFM4 by a substance present in plasma as exogenous added OLFM4 is readily detected in such plasma. High levels of OLFM4 were found both in normals and in patients with verified colorectal cancer and bare no relation to the presence of cancer. Thus, determination of OLFM4 in plasma cannot be used as a biomarker for colorectal cancer.

The source of OLFM4 present in plasma is not clear. While the amount of OLFM4 present in neutrophils is substantial, indicating production of 10 mg/day, there was no difference in the retention of OLFM4 during biosynthesis in neutrophil precursors from bone marrow of a plasma OLFM4‐high person and a plasma OLFM4‐low person, indicating that the difference in OLFM4 levels in plasma cannot be explained by secretion from the bone marrow. The gradient of OLFM4 in bone marrow plasma and blood plasma also argues against the bone marrow as the direct source of plasma OLFM4. We cannot, of course, rule out that cells in the GI tract are the source of plasma OLFM4, but find this highly unlikely as we find a uniform staining of cells among the sections examined and also find that plasma levels do not correlate with the presence or absence of cancers in the GI tract. In agreement with this, another study correlating plasma levels of OLFM4 with cancers concluded that plasma concentration of OLFM4 did not correlate well with OLFM4 expression in the tumours as determined by immunohistochemistry, although that study did not identify patients with high plasma levels as we observe 23. Our studies also indicate that OLFM4 is not synthesized by the liver, and we did not find any difference in the low levels of expression (if any) among 42 individuals. It is of interest that OLFM4 was demonstrated in benign prostate tissue, but not in prostate adenocarcinomas.

We find that OLFM4 is highly sensitive to proteolysis. Our current hypothesis is therefore that the differences in OLFM4 levels in plasma may be related to individual differences in the susceptibility of OLFM4 to escape degradation when neutrophils decease as part of their normal life cycle. Production of 10 mg OLFM4/day would support a plasma level of 3–4 μg/ml plasma depending of the half‐life of OLFM4 in plasma. This hypothesis is not easily tested, but if proven correct, might open for novel insight into the fate of neutrophils after exiting circulation, an issue that is still a matter of debate.

## Conflicts of interest

The authors confirm that there are no conflicts of interest.

## Supporting information


**Figure S1** Immunohistochemistry of human tissues using anti‐OLFM4 clone #49 as primary antibody.

**Figure S2** Immunohistochemistry of prostate tissue.

**Figure S3** Immunohistochemistry of different histopathological subtypes of colorectal tumors.

**Table S1** Basic characteristics of patients in figure 4B.

## References

[1] Zhang J , Liu WL , Tang DC , *et al* Identification and characterization of a novel member of olfactomedin‐related protein family, hGC‐1, expressed during myeloid lineage development. Gene. 2002; 283: 83–93. 11867215 10.1016/s0378-1119(01)00763-6

[2] Rosenbauer F , Wagner K , Zhang P , *et al* pDP4, a novel glycoprotein secreted by mature granulocytes, is regulated by transcription factor PU.1. Blood. 2004; 103: 4294–301. 14962908 10.1182/blood-2003-08-2688

[3] Clemmensen SN , Bohr CT , Rorvig S , *et al* Olfactomedin 4 defines a subset of human neutrophils. J Leukoc Biol. 2012; 91: 495–500. 22187488 10.1189/jlb.0811417 PMC3289394

[4] Welin A , Amirbeagi F , Christenson K , *et al* The human neutrophil subsets defined by the presence or absence of OLFM4 both transmigrate into tissue *in vivo* and give rise to distinct NETs *in vitro* . PLoS ONE. 2013; 8: e69575. 23922742 10.1371/journal.pone.0069575 PMC3726694

[5] Zhang X , Huang Q , Yang Z , *et al* GW112, a novel antiapoptotic protein that promotes tumor growth. Cancer Res. 2004; 64: 2474–81. 15059901 10.1158/0008-5472.can-03-3443

[6] Liu W , Liu Y , Wang R , *et al* Olfactomedin 4 is essential for superoxide production and sensitizes oxidative stress‐induced apoptosis in neutrophils. ASH Annu Meet Abstr. 2009; 114: 1356.

[7] Liu W , Yan M , Liu Y , *et al* Olfactomedin 4 inhibits cathepsin C‐mediated protease activities, thereby modulating neutrophil killing of Staphylococcus aureus and Escherichia coli in mice. J Immunol. 2012; 189: 2460–7. 22844115 10.4049/jimmunol.1103179 PMC3424379

[8] Liu W , Yan M , Sugui JA , *et al* Olfm4 deletion enhances defense against Staphylococcus aureus in chronic granulomatous disease. J Clin Invest. 2013; 123: 3751–5. 23908114 10.1172/JCI68453 PMC3754258

[9] Sorensen OE , Clemmensen SN , Dahl SL , *et al* Papillon‐Lefevre syndrome patient reveals species‐dependent requirements for neutrophil defenses. J Clin Invest. 2014; 124: 4539–48. 25244098 10.1172/JCI76009 PMC4191054

[10] Adkison AM , Raptis SZ , Kelley DG , *et al* Dipeptidyl peptidase I activates neutrophil‐derived serine proteases and regulates the development of acute experimental arthritis. J Clin Invest. 2002; 109: 363–71. 11827996 10.1172/JCI13462 PMC150852

[11] van der Flier LG , Haegebarth A , Stange DE , *et al* OLFM4 is a robust marker for stem cells in human intestine and marks a subset of colorectal cancer cells. Gastroenterology. 2009; 137: 15–7. 19450592 10.1053/j.gastro.2009.05.035

[12] Schuijers J , van der Flier LG , van Es J , *et al* Robust cre‐mediated recombination in small intestinal stem cells utilizing the olfm4 locus. Stem Cell Reports. 2014; 3: 234–41. 25254337 10.1016/j.stemcr.2014.05.018 PMC4175542

[13] Gersemann M , Becker S , Nuding S , *et al* Olfactomedin‐4 is a glycoprotein secreted into mucus in active IBD. J Crohns Colitis. 2012; 6: 425–34. 22398066 10.1016/j.crohns.2011.09.013

[14] Chin KL , Aerbajinai W , Zhu J , *et al* The regulation of OLFM4 expression in myeloid precursor cells relies on NF‐kappaB transcription factor. Br J Haematol. 2008; 143: 421–32. 18764868 10.1111/j.1365-2141.2008.07368.x

[15] Liu W , Yan M , Liu Y , *et al* Olfactomedin 4 down‐regulates innate immunity against *Helicobacter pylori* infection. Proc Natl Acad Sci USA. 2010; 107: 11056–61. 20534456 10.1073/pnas.1001269107 PMC2890768

[16] Dassen H , Punyadeera C , Delvoux B , *et al* Olfactomedin‐4 regulation by estrogen in the human endometrium requires epidermal growth factor signaling. Am J Pathol. 2010; 177: 2495–508. 21048224 10.2353/ajpath.2010.100026 PMC2966806

[17] Koshida S , Kobayashi D , Moriai R , *et al* Specific overexpression of OLFM4(GW112/HGC‐1) mRNA in colon, breast and lung cancer tissues detected using quantitative analysis. Cancer Sci. 2007; 98: 315–20. 17270020 10.1111/j.1349-7006.2006.00383.x PMC11159027

[18] Liu W , Zhu J , Cao L , *et al* Expression of hGC‐1 is correlated with differentiation of gastric carcinoma. Histopathology. 2007; 51: 157–65. 17650212 10.1111/j.1365-2559.2007.02763.x

[19] Chen L , Li H , Liu W , *et al* Olfactomedin 4 suppresses prostate cancer cell growth and metastasis *via* negative interaction with cathepsin D and SDF‐1. Carcinogenesis. 2011; 32: 986–94. 21470957 10.1093/carcin/bgr065 PMC3128558

[20] Park KS , Kim KK , Piao ZH , *et al* Olfactomedin 4 suppresses tumor growth and metastasis of mouse melanoma cells through downregulation of integrin and MMP genes. Mol Cells. 2012; 34: 555–61. 23161172 10.1007/s10059-012-0251-7 PMC3887829

[21] Karagiannis GS , Pavlou MP , Saraon P , *et al* In‐depth proteomic delineation of the colorectal cancer exoproteome: mechanistic insight and identification of potential biomarkers. J Proteomics. 2014; 103: 121–36. 24681409 10.1016/j.jprot.2014.03.018

[22] de Wit M , Kant H , Piersma SR , *et al* Colorectal cancer candidate biomarkers identified by tissue secretome proteome profiling. J Proteomics. 2014; 99: 26–39. 24418523 10.1016/j.jprot.2014.01.001

[23] Oue N , Sentani K , Noguchi T , *et al* Serum olfactomedin 4 (GW112, hGC‐1) in combination with Reg IV is a highly sensitive biomarker for gastric cancer patients. Int J Cancer. 2009; 125: 2383–92. 19670418 10.1002/ijc.24624

[24] Kohler G , Milstein C . Continuous cultures of fused cells secreting antibody of predefined specificity. Nature. 1975; 256: 495–7. 1172191 10.1038/256495a0

[25] Borregaard N , Heiple JM , Simons ER , *et al* Subcellular localization of the b‐cytochrome component of the human neutrophil microbicidal oxidase: translocation during activation. J Cell Biol. 1983; 97: 52–61. 6408102 10.1083/jcb.97.1.52 PMC2112494

[26] Kjeldsen L , Sengeløv H , Lollike K , *et al* Isolation and characterization of gelatinase granules from human neutrophils. Blood. 1994; 83: 1640–9. 8123855

[27] Clemmensen SN , Udby L , Borregaard N . Subcellular fractionation of human neutrophils and analysis of subcellular markers. Methods Mol Biol. 2014; 1124: 53–76. 24504946 10.1007/978-1-62703-845-4_5

[28] Clemmensen SN , Jacobsen LC , Rorvig S , *et al* Alpha‐1‐antitrypsin is produced by human neutrophil granulocytes and their precursors and liberated during granule exocytosis. Eur J Haematol. 2011; 86: 517–30. 21477074 10.1111/j.1600-0609.2011.01601.x

[29] Bordier C . Phase separation of integral membrane proteins in Triton X‐114 solution. J Biol Chem. 1981; 256: 1604–7. 6257680

[30] Liu W , Chen L , Zhu J , *et al* The glycoprotein hGC‐1 binds to cadherin and lectins. Exp Cell Res. 2006; 312: 1785–97. 16566923 10.1016/j.yexcr.2006.02.011

[31] Grover PK , Hardingham JE , Cummins AG . Stem cell marker olfactomedin 4: critical appraisal of its characteristics and role in tumorigenesis. Cancer Metastasis Rev. 2010; 29: 761–75. 20878207 10.1007/s10555-010-9262-z

[32] Kjeldsen L , Johnsen AH , Sengeløv H , *et al* Isolation and primary structure of NGAL, a novel protein associated with human neutrophil gelatinase. J Biol Chem. 1993; 268: 10425–32. 7683678

[33] Storm L , Christensen IJ , Jensenius JC , *et al* Evaluation of complement proteins as screening markers for colorectal cancer. Cancer Immunol Immunother. 2015; 64: 41–50. 25261356 10.1007/s00262-014-1615-y PMC11028411

[34] Mary JY . Normal human granulopoiesis revisited. II. Bone marrow data. Biomed Pharmacother. 1985; 39: 66–77. 3893558

[35] Scott EW , Simon MC , Anastasi J , *et al* Requirement of transcription factor PU.1 in the development of multiple hematopoietic lineages. Science. 1994; 265: 1573–7. 8079170 10.1126/science.8079170

