# Supplementary figures and images for: Plasma levels of OLFM4 in normals and patients with gastrointestinal cancer
Source: J Cell Mol Med. 2015 Sep 28;19(12):2865–73. doi: 10.1111/jcmm.12679 (PMC4687705; doi:10.1111/jcmm.12679)

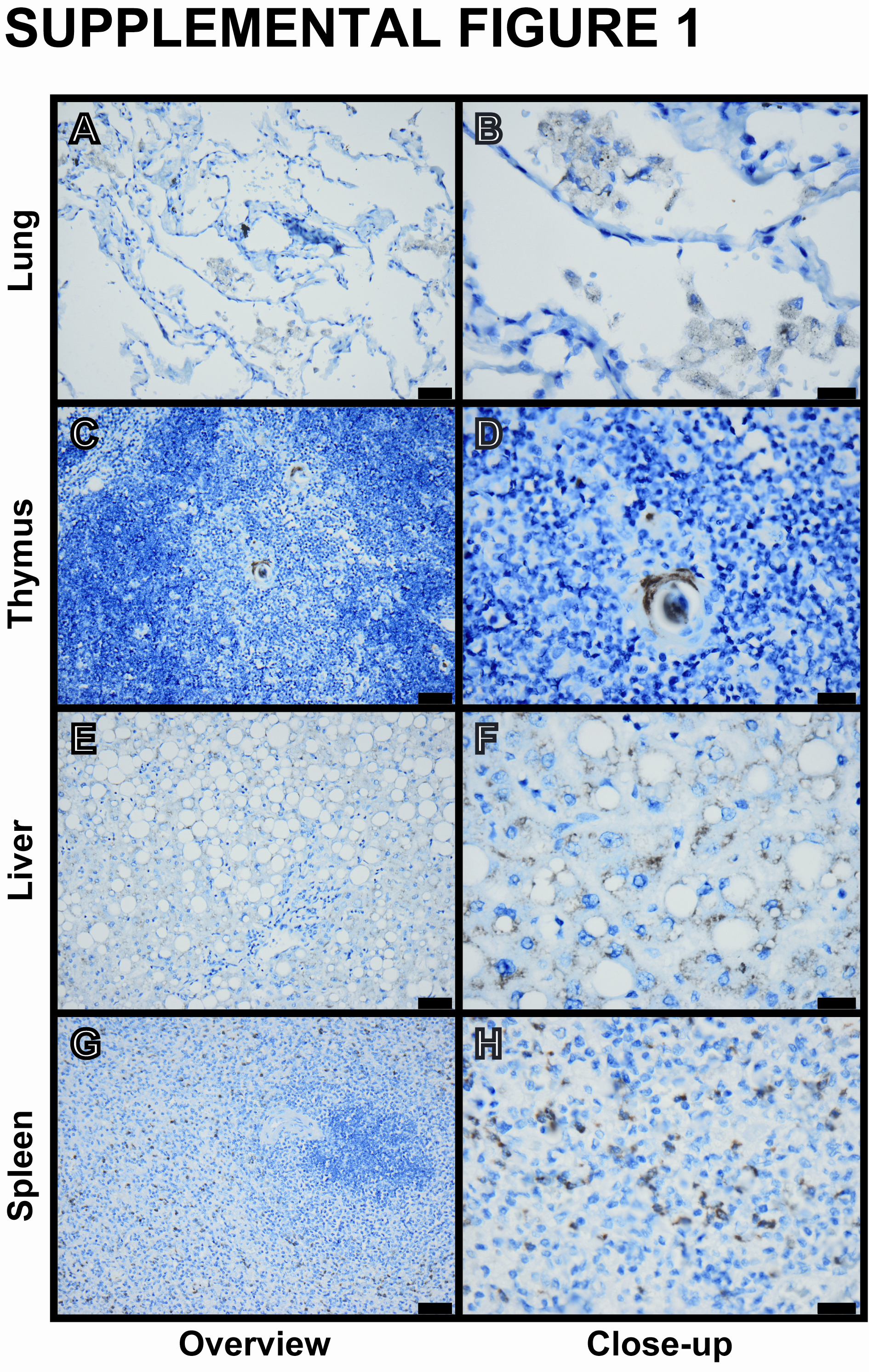

Supplement: Supplementary file 1 — Figure S1 Immunohistochemistry of human tissues using anti‐OLFM4 clone #49 as primary antibody. [file JCMM-19-2865-s001.tif]

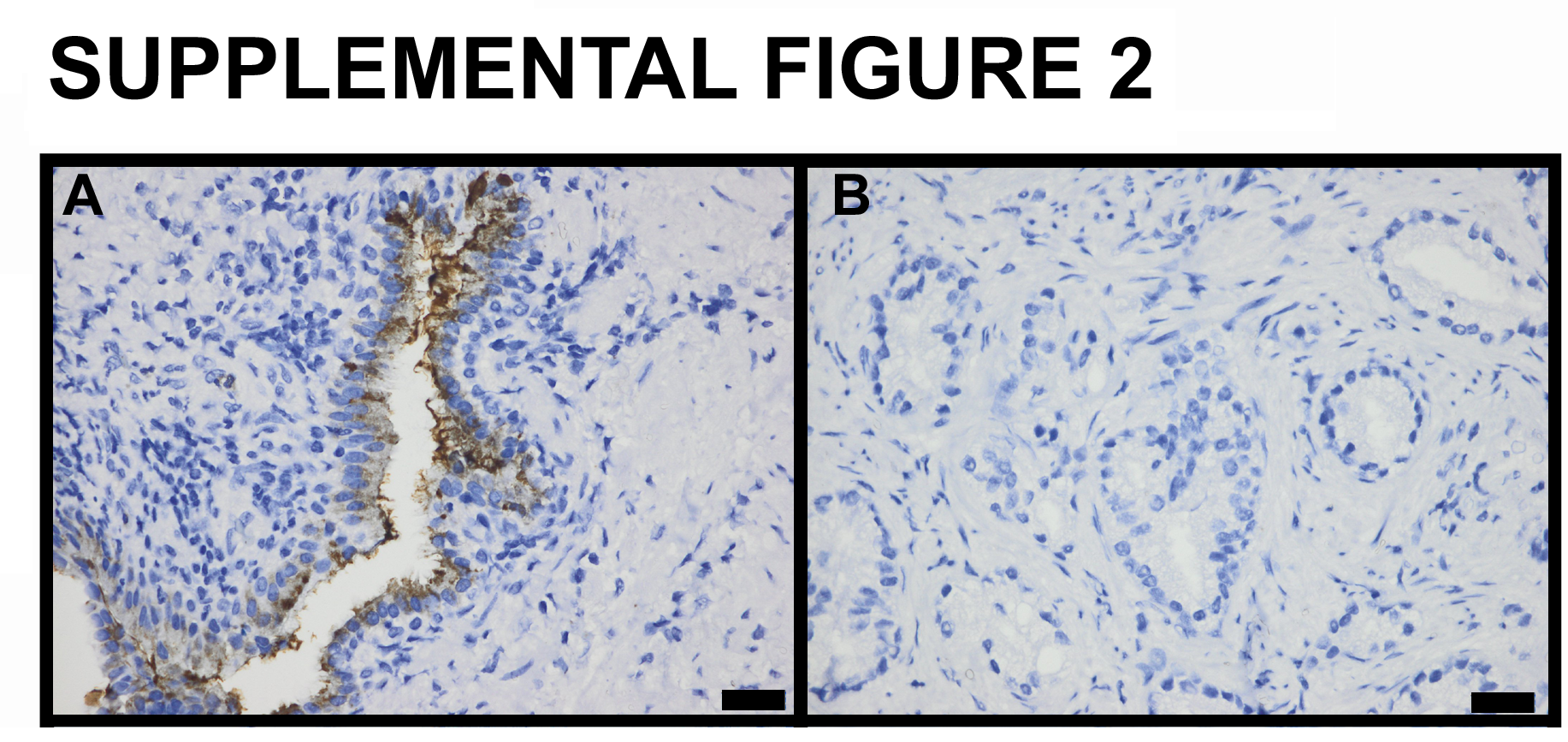

Supplement: Supplementary file 2 — Figure S2 Immunohistochemistry of prostate tissue. [file JCMM-19-2865-s002.tif]

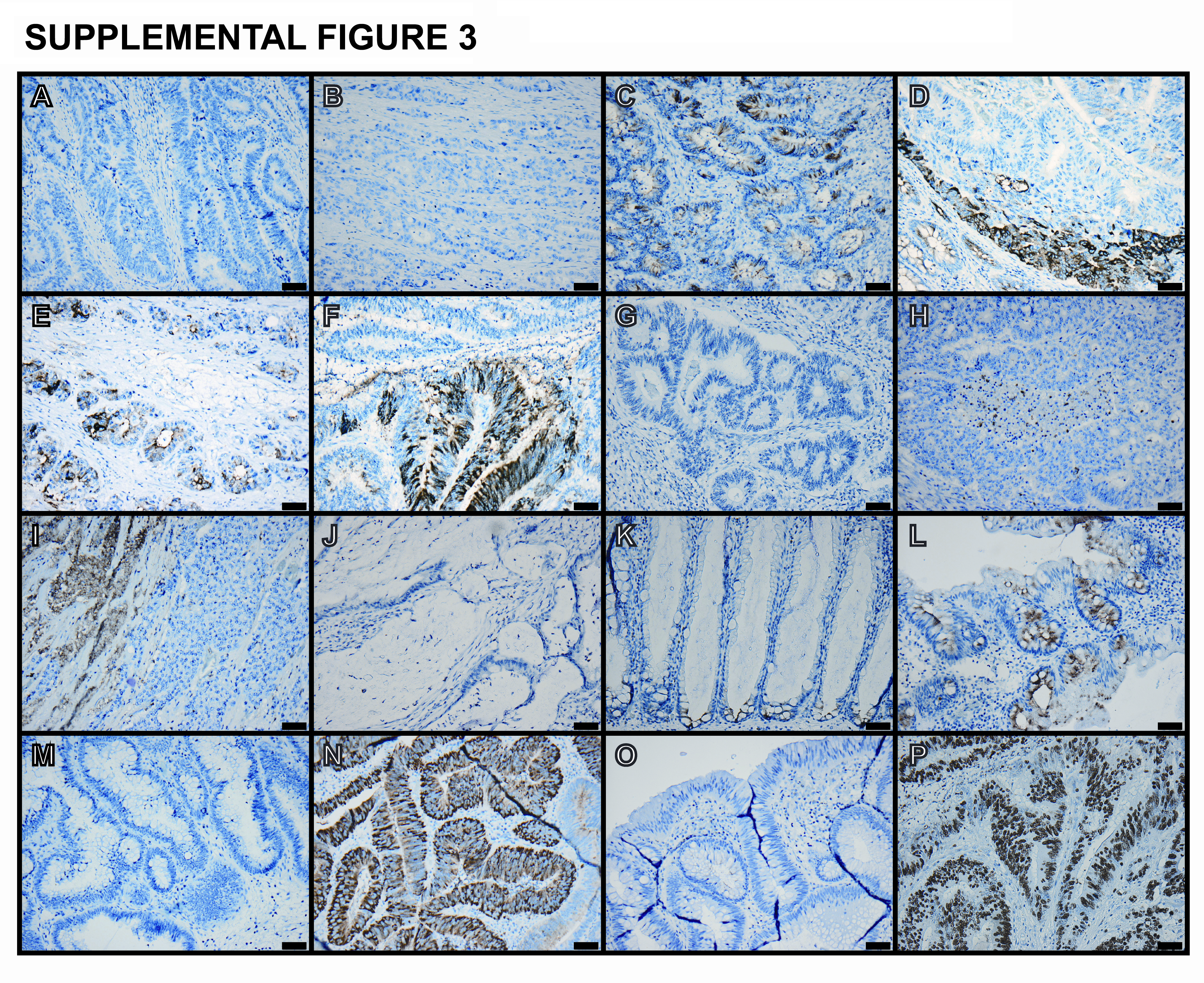

Supplement: Supplementary file 3 — Figure S3 Immunohistochemistry of different histopathological subtypes of colorectal tumors. [file JCMM-19-2865-s003.tif]

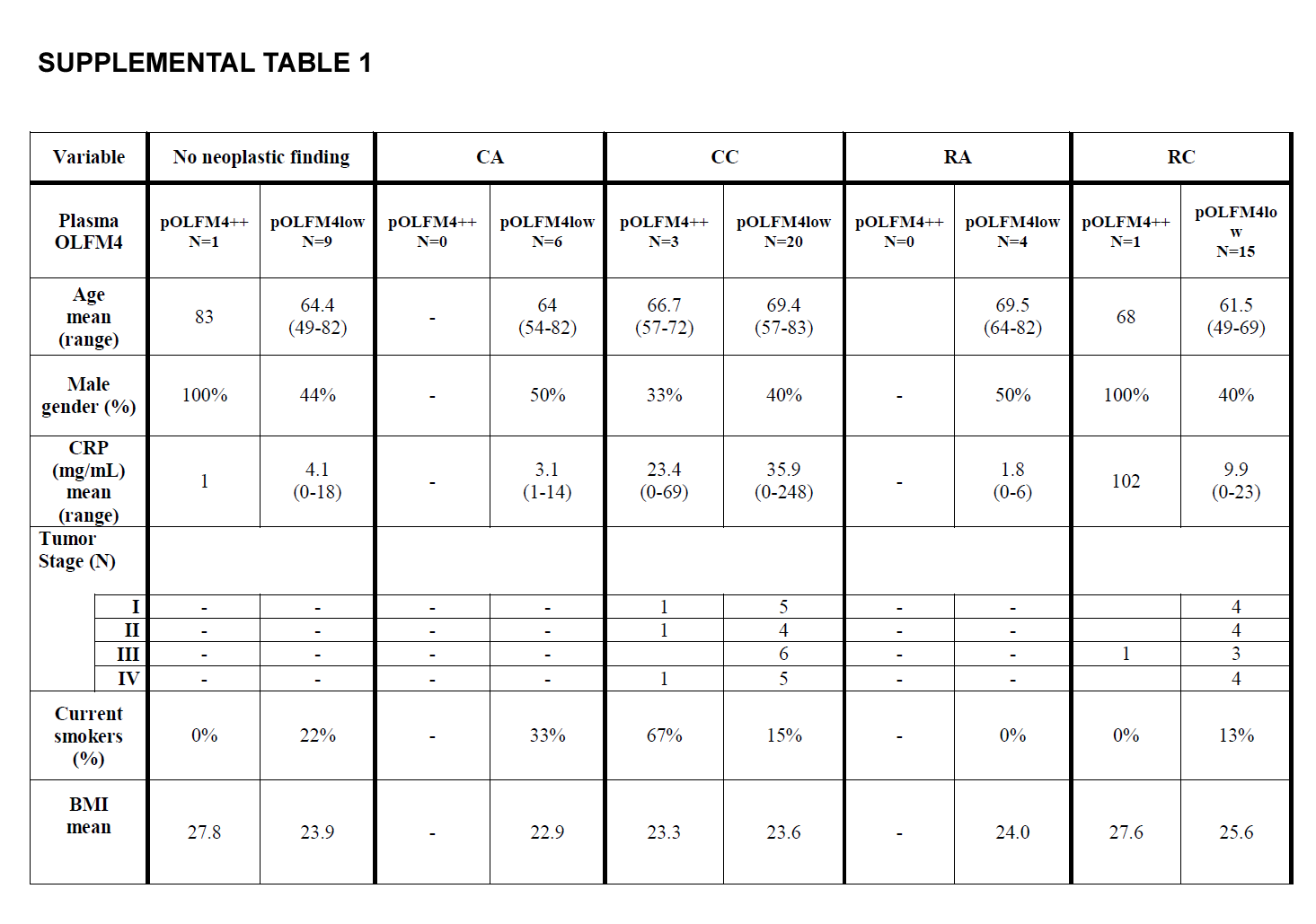

Supplement: Supplementary file 4 — Table S1 Basic characteristics of patients in figure 4B. [file JCMM-19-2865-s004.tif]
